# Simulated nanoindentation into single-phase fcc Fe$_{x}$Ni$_{1-x}$ alloys predicts maximum hardness for equiatomic stoichiometry

**DOI:** 10.1038/s41598-023-36899-3

**Published:** 2023-06-16

**Authors:** Iyad Alabd Alhafez, Orlando R. Deluigi, Diego Tramontina, Carlos J. Ruestes, Eduardo M. Bringa, Herbert M. Urbassek

**Affiliations:** 1 Physics Department and Research Center OPTIMAS, University Kaiserslautern-Landau, Erwin-Schrödinger-Straße, 67663 Kaiserslautern, Germany; 2 Institute of Applied Mechanics, Clausthal University of Technology, Adolph-Roemer Str. 2A, 38678 Clausthal-Zellerfeld, Germany; 3 CONICET and Facultad de Ingenería, Universidad de Mendoza, 5500 Mendoza, Argentina; 4 IMDEA Materials Institute, C/Eric Kandel 2, 28906 Getafe, Madrid Spain; 5 Instituto Interdisciplinario de Ciencias Básicas (ICB), Universidad Nacional de Cuyo UNCuyo-CONICET, Facultad de Ciencias Exactas y Naturales, Padre Contreras 1300, 5500 Mendoza, Argentina; 6 Centro de Nanotecnología Aplicada, Universidad Mayor, Santiago, Chile

**Keywords:** Materials science, Physics

## Abstract

We investigate by molecular dynamics simulation the mechanical behavior of concentrated alloys under nanoindentation for the special example of single-phase fcc Fe$_{x}$Ni$_{1-x}$ alloys. The indentation hardness is maximum for the equiatomic alloy, $x=0.5$. This finding is in agreement with experimental results on the strength of these alloys under uniaxial strain. We explain this finding with the increase of the unstable stacking fault energy in the alloys towards $x=0.5$. With increasing Fe content, loop emission from the plastic zone under the indenter becomes less pronounced and the plastic zone features a larger fraction of screw dislocation segments; simultaneously, the length of the dislocation network and the number of atoms in the stacking faults generated in the plastic zone increase. However, the volume of twinned regions in the plastic zone is highest for the elemental solids and decreases for the alloys. This feature is explained by the fact that twinning proceeds by the glide of dislocations on adjacent parallel lattice planes; this concerted motion is less efficient in the alloys. Finally, we find that surface imprints show increasing pile-up heights with increasing Fe content. The present results will be of interest for hardness engineering or generating hardness profiles in concentrated alloys.

## Introduction

The Fe-Ni system is of interest in various areas of science and technology, ranging from steels over magnetic materials to the geophysics of planetary interiors and meteorites^1^. At high temperatures, above 1183 K, the two elements are completely miscible in the form of random alloys with fcc structure. At lower temperature, the phase diagram is richer and features a bcc phase as well as intermetallic compounds^2^. Since Fe and Ni are important basis elements for steels, the properties of the FeNi system are also often considered in the context of wider classes of alloys, in particular the ternary FeNiCr system.

When prepared in their fcc phase, FeNi alloys belong to the class of single-phase concentrated solid-solution alloys, which have received great interest because their mechanical properties may deviate strongly from that of conventional dilute alloys^3–5^. High-entropy alloys form another, intensely investigated, member of the class of concentrated alloys^6–9^.

The mechanical and thermal properties of Fe-Ni alloys have been investigated using ab initio techniques and by molecular dynamics (MD) simulation. Mishin *et al.*^10^ studied the phase diagram using DFT. The martensitic transformation from the high-temperature fcc to the low-temperature bcc phase was explored using MD simulation^11,12^. Plastic properties, in particular dislocation glide^13–15^ and also work hardening^16^ were explored by MD. Basic dislocation properties, in particular the stacking fault energy (SFE) were investigated using DFT^17^. Diffusion processes in Fe$_{50}$Ni$_{50}$ random alloys were studied by Osetsky *et al.*^18^ using MD and kinetic Monte Carlo as an example of the sluggish diffusion in concentrated alloys^19^. The evolution of extended defects, such as dislocation loops, under irradiation was studied by experiment and MD^20,21^. The influence of stress on radiation-induced properties was studied by simulations in Ref.^22^. The effect of fluctuations of the SFE on the strengthening in concentrated fcc alloys was investigated theoretically^23^. Nöhring and Curtin^24^ use MD to study cross-slip in fcc alloys and emphasize the role of local stoichiometry fluctuations on activation energy barriers. Also the interaction of dislocations with twin boundaries were studied in these alloys by MD^25^.

In the present paper, we study the indentation hardness of random Fe$_{x}$Ni$_{1-x}$ solid solutions with fcc structure. While these crystals do not exist in the equilibrium phase diagram at low temperatures, they can be produced by drop casting to obtain single crystals^26,27^. MD simulation allows us to perform exactly the same indentation on a series of Fe$_{x}$Ni$_{1-x}$ crystals to monitor the differences in hardness in these crystals and correlate them with the dislocation forests generated. In addition, by a study of the generalized stacking fault energy curves, we can correlate the differences in hardness of these samples with the unstable stacking-fault energy and thus obtain insight into the microphysics of the hardness of these alloys.

## Methods

We study the behavior of random Fe$_{x}$Ni$_{1-x}$ fcc alloys ($x=0.25$, 0.5 and 0.75) under indentation and compare it to the behavior of elemental fcc-Fe and Ni. Since at high temperatures, these alloys have fcc structure, and Ni is fcc, we also employ Fe in the fcc structure. The potential by Bonny *et al.*^28^ is used for modeling these metals and alloys. The authors of this potential took care to reproduce both elastic properties and stacking fault energies (SFE) for Ni and fcc-Fe. This potential has been used previously to study the behavior of, among others, dislocation properties^14,29^, diffusion^18^, dislocation interaction^30^ and work hardening^16^ in fcc-Fe, in Fe$_{x}$Ni$_{1-x}$ alloys and in austenitic FeNiCr alloys. A later extension^31^ was ascertained to show little differences on dislocation properties, but focused on a better description of point defect properties at the expense of the elastic properties.

The random alloys are created by setting Fe and Ni randomly on the sites of an fcc lattice with probabilities chosen according to the desired stoichiometries. The crystals have a (100) surface, lateral extensions of around 66.4 nm and depths of 50.3 nm. They contain around 20.8 million atoms. Then the samples are relaxed in an NPT ensemble in two steps; first, using periodic boundary conditions in all directions, and then with a free surface to allow for indentation. Their final temperature is around 1 mK and they show low stress components around $10^{-4}$ GPa. Note that our sample is big enough to avoid any passage of dislocations through the lateral boundaries; such passages would be unphysical and lead to self-interaction of the plastic zone.

**Figure 1:**
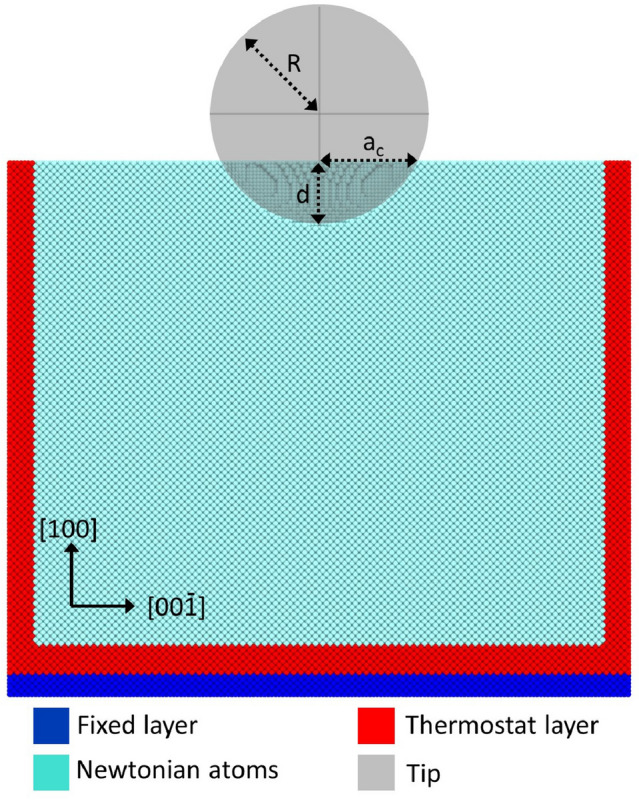
Schematics of the simulation setup. The indenter geometry is characterized by its radius *R*, the indentation depth *d* and the contact radius $a_c$.

Two atomic layers of the substrate at the bottom and the lateral sides are fixed to prevent the whole substrate from any translational movement. Figure 1 shows a schematics of the simulation setup. The next four layers are kept at a temperature of 1 mK by a velocity-scaling thermostat. Such a low temperature is chosen to minimize thermal vibrations and help in the identification of defects. We note that small quantitative differences in the indentation results between the 1-mK indent and an indent at room temperature can be expected, but no changes in the plasticity mechanisms^32,33^. Thus, a previous simulation study^34^ on bcc-Fe compared a 0-K and a 300-K indent and demonstrated a systematic increase in dislocation length by 5.5% accompanied by a decrease in indentation hardness by 12%.

The indenter is modeled as a repulsive sphere^35^. The interaction potential between the indenter and the substrate atoms is limited to distances $r<R$, where *R* is the indenter radius; in this study we use $R = 10$ nm. The indenter interacts with the substrate atoms by the potential

1 $$\begin{aligned} V(r) = \left\{ \begin{array}{ll} k(R-r)^3, &{} r<R, \\ 0, &{} r\ge R, \end{array} \right. \end{aligned}$$

where *r* is the distance of a substrate atom to the center of the indenter. The indenter stiffness has been set to $k= 10$ eV/Å$^3$^35,36^.

The indenter is initially positioned immediately above the surface; at time $t=0$ it starts moving perpendicular to the target with a velocity of 20 m/s. We penetrate to a final depth of $d=4$ nm, which is reached at $t=200$ ps. Then the indenter is held fixed for a time of 100 ps, until it recedes with a velocity of 20 m/s.

We use the open-source LAMMPS code^37^ with a constant time step of 1 fs to perform the simulations. The free software tool OVITO^38^, which also includes the Dislocation Extraction Algorithm (DXA) tool needed for dislocation analysis^39–41^, is employed to visualize the atomistic configurations, to identify the dislocations and to measure the total length of the dislocation lines, $L_{\textrm{disl}}$. Twin boundaries and dislocation junctions were identified with the Crystal Analysis Tool CAT^40,42,43^.

In order to extract the twinned regions, we use the orientation quaternions obtained from the Polyhedral Template Matching (PTM) filter^44^ available in OVITO, and transform them to vectors in Rodrigues space^45^. We calculate the misorientation angle $\theta$ for each atom from the dot product between the Rodrigues vector of that atom at a given frame and the vector at the initial frame^46^. Plasticity, including twinning, dislocations and stacking faults, leads to rotations with $\theta$ greater than $15^\circ$. We then compute the coordination up to the fourth-nearest neighbors (cutoff at 0.505 nm). By filtering out atoms with coordination smaller than 31 and recalculating coordination with first-nearest neighbors (cutoff taken as 0.27 nm), we filter out partial dislocations, stacking faults and dislocation junctions, leaving only the twinned volume with a thickness greater than 2 layers.

## Results

### Stable and unstable stacking fault energy

**Figure 2:**
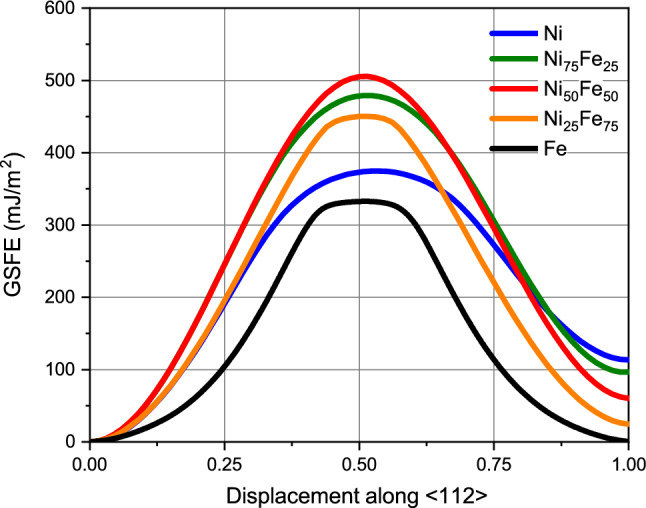
Generalized stacking fault energy, GSFE, of Fe, Ni and several random Fe$_{x}$Ni$_{1-x}$ alloys calculated with the Bonny potential^28^. Abscissa axis in units of $a_0/\sqrt{6}$.

The quantification of plastic deformation in crystals requires the discussion of the stacking fault energy (SFE). Its definition for fcc crystals is conveniently based on the generalized stacking fault energy curves displayed in Fig. 2. These monitor the energy increase upon rigidly shifting two adjacent (111) planes in an fcc crystal along the $[11{\bar{2}}]$ direction^36,47,48^. The minimum at $a_0/\sqrt{6}$ ($a_0$ is the lattice constant) gives the SFE. The maximum at around half this displacement that has to be crossed to form a stacking fault is denoted as the unstable stacking fault energy (USFE). The values of the SFE and USFE of Fe$_{x}$Ni$_{1-x}$ alloys are assembled in Table 1.

The SFE of fcc-Fe is very small; Bonny *et al.*^28^ argue that this is in line with extrapolations of experimental Fe$_{x}$Ni$_{1-x}$ data to low *x*. Table 1 shows that the SFE decreases approximately linearly with Fe content *x*, cf. Fig. 3 of Ref.^28^. The USFE shows a more complex behavior as it assumes approximately similar values for the elements, but exhibits a maximum for intermediate stoichiometries.

The SFE of elemental Ni has been explored both by experiment (128 mJ/m$^2$ according to Ref.^28^) and DFT (133 mJ/m$^2$, see Ref.^17^). Experimental data of Ref.^49^ on fcc-Fe$_{x}$Ni$_{1-x}$ find a minimum in the SFE for 40% Fe content at 102 mJ/m$^2$, but the authors are aware of problems caused by the partial martensitic transformation to bcc at low Fe content and also find a too high SFE of > 200 mJ/m$^2$ for pure Ni. More recently, Refs.^50,51^ reported an experimental value of 79 mJ/m$^2$ for equiatomic Fe$_{50}$Ni$_{50}$. Reference^14^ reports the SFE of NiFe in the Bonny potential to be 113 mJ/m$^2$, in close agreement with our findings. Reference^17^ finds a value of 105 mJ/m$^2$ for equiatomic FeNi from DFT calculations, which is somewhat larger than the value obtained for the Bonny potential, 63 mJ/m$^2$ (Table 1), other empirical simulation studies find a value of 31 mJ/m$^2$, see Ref.^52^, and 28 mJ/m$^2$, Ref.^53^. Finally, we mention that the strong and monotonic increase of the SFE with Ni content is in agreement with the vast body of knowledge on the composition-dependence of SFEs in austenitic stainless steels^54^.

Ab-initio data for the USFE are more rare. Ref.^17^ gives 281 mJ/m$^2$ for pure Ni and around 230 mJ/m$^2$ for NiFe, somewhat below that of pure Ni. In the Bonny *et al.* potential^28^, Fe$_{50}$Ni$_{50}$ has a higher USFE than pure Ni, and this is retained in related embedded-atom-method (EAM) potentials^31,55^, unlike the case for a recent modified EAM (MEAM) potential^56^.

We note that in some previous studies of Fe$_{x}$Ni$_{1-x}$ alloys, the Choi potential^57^, which was developed for the simulation of CoCrFeMnNi high-entropy alloys, was used^58^. To our opinion, this potential is not well suited to studying the Fe$_{x}$Ni$_{1-x}$ subsystem, since (i) USFE decreases with increasing *x* monotonically, reaching only 100 mJ/m$^2$ for pure Fe; (ii) the potential for pure Fe gives even a negative SFE of around $-100$ mJ/m$^2$. These features enhance prolific dislocation production for Fe-rich Fe$_{x}$Ni$_{1-x}$ alloys. Indeed, the original paper^57^, Fig. 2, already showed that Fe$_{50}$Ni$_{50}$ has a significantly decreased ultimate tensile strength and yield strength compared to Ni.

**Table 1:** SFE and USFE for the Bonny potential.^28^.

	Ni	Fe$_{25}$Ni$_{75}$	Fe$_{50}$Ni$_{50}$	Fe$_{75}$Ni$_{25}$	Fe
SFE (mJ/m$^2$)	113	99	63	25	0.2
USFE (mJ/m$^2$)	375	478	507	453	328
SFE/USFE	0.301	0.207	0.125	0.055	0.001

### Indentation hardness

**Figure 3:**
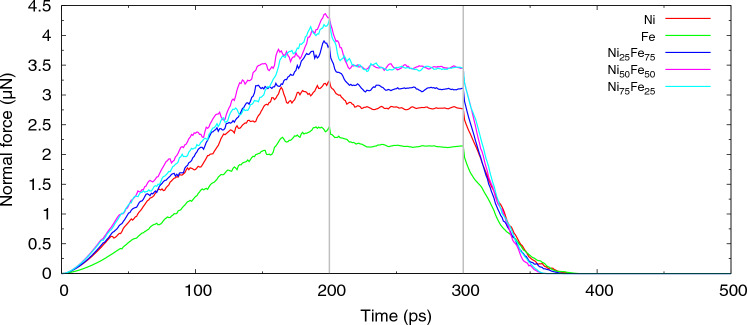
Evolution of the normal force in Fe, Ni and several random Fe$_{x}$Ni$_{1-x}$ alloys. Indentation proceeds until time 200 ps; the indenter is held constant until 300 ps and then retracted. The vertical lines indicate these times.

**Figure 4:**
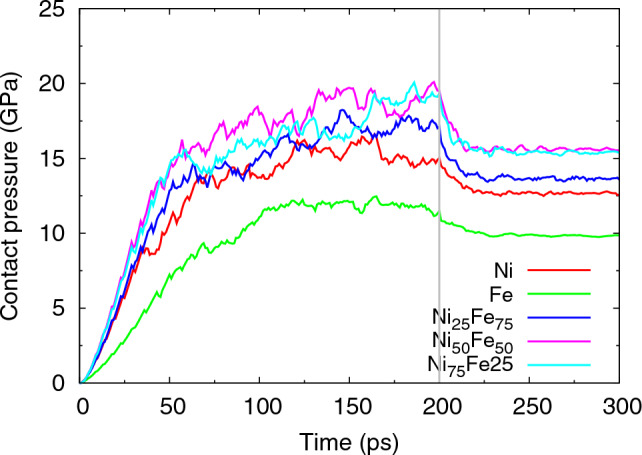
Evolution of the contact pressure during the indent and hold phases.

Figure 3 displays the immediate outcome of the indentation simulation, viz. the evolution of the indentation force with time. During the indentation phase, the force increases; the fluctuations visible are caused by bursts of dislocation emission, which temporarily release the stress under the indenter and hence decrease the normal force. During the hold phase, the normal force sinks, since relaxation and re-organization of the dislocation network formed under the indenter help to relax the material. Finally, during the retraction phase, the force quickly vanishes as the indenter is withdrawn from the sample. Figure 4 plots the contact pressure during the indent and hold phases from the ratio of the normal force to the projected contact area. As the contact area changes strongly with indentation depth, force and pressure have a different depth dependence during the indentation phase.

**Figure 5:**
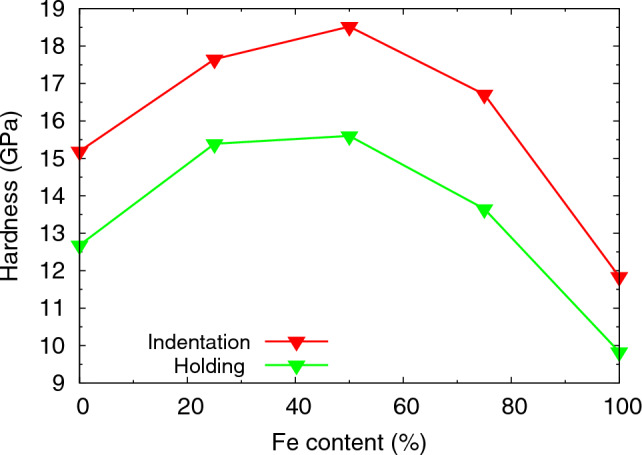
Dependence of indentation hardness on Fe content of elemental Fe and Ni and several random Fe$_{x}$Ni$_{1-x}$ alloys on Fe content. Data are averages over the last 2 nm of the indentation stage (red) and the last 50 ps during hold stage (green).

Figures 3 and 4 give the important information that more force is needed to indent the alloys than the pure elements. We quantify this finding by plotting the indentation hardness *H* in Fig. 5. Here, we determine *H* as the average contact pressure during the last 2 nm of indentation. As the force decreased during the hold phase, cf. Fig. 3, we also calculated the average contact pressure during the hold phase (averaged over the last 50 ps of the hold); it shows the same feature of a maximum hardness for the alloys.

**Figure 6:**
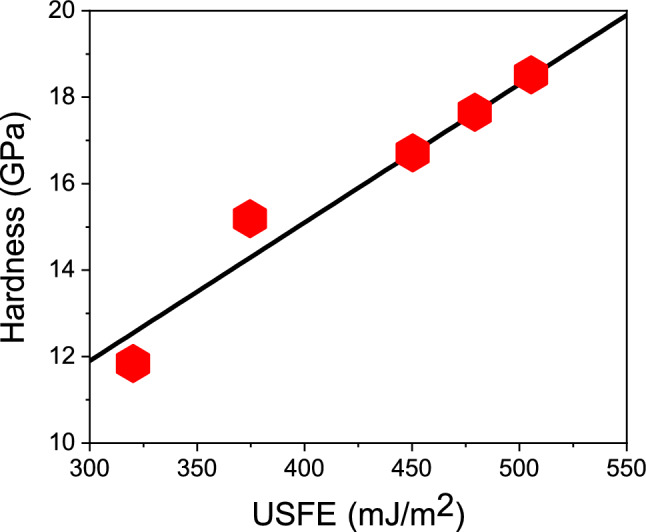
Correlation of the indentation hardness and the USFE for elemental Fe and Ni and several random Fe$_{x}$Ni$_{1-x}$ alloy samples. Line to guide the eye.

An explanation for the increased hardness of alloys as compared to that of the elemental materials can be obtained by a consideration of the USFE, Table 1, which features the same behavior, cf. Fig. 6. Indeed, as the USFE quantifies the energy barrier that needs to be surpassed for stacking fault creation, it correlates with the material hardness. Rice^59^ proposed that the ratio SFE/USFE can be used as a measure of ductility. By tuning the USFE in the interatomic potential, Ref.^60^ demonstrated that hardness increases with USFE. References^61,62^ demonstrated in a computational study which compared various fcc metals that both flow stress and stress at first dislocation emission in tensile straining correlate well with the USFE, corroborating earlier work on the role of the USFE on mechanical properties in fcc metals^63–66^. Recently, Jarlöv *et al.*^67^ demonstrated a linear correlation between the USFE and the yield stress in CrFeCoNi high-entropy alloys using MD simulation of uniaxial tensile deformation. Recently, the plastic yielding of Co$_x$Ni$_{1-x}$ nanoparticles under indentation was also related to changes in the USFE^68^.

Experimentally, the indentation hardness of single-phase random-solution fcc Fe$_{x}$Ni$_{1-x}$ alloys has been measured in several studies. Schwaiger *et al.*^69^ find a hardness increase for nanocrystalline Ni upon the addition of 2.6 and 5.6% Fe. Kurpaska *et al.*^58^ performed experiments and simulations of nanoindentation into Ni and Fe$_{x}$Ni$_{1-x}$ alloys ($x=0.2$ and 0.5) and observed the alloys to be harder than the pure Ni. In these experiments, $x=0.2$ and $x=0.5$ have approximately similar hardness, as in our hardness values after tip retraction (Fig. 5). Finally, Yang *et al.*^70^ determined the indentation hardness of Fe$_{50}$Ni$_{50}$ to be around 30% higher than of pure Ni, in reasonable agreement with our results (Fig. 5). This paper also reports a strong increase of the dislocation density in Fe$_{50}$Ni$_{50}$, which is qualitatively in agreement with our simulations, Table 2, but quantitatively even stronger (100% increase).

**Table 2:** Characteristics of the plastic zone after indent and after retraction of the tip. $L_{\textrm{disl}}$: total dislocation length within plastic zone; $R_{\textrm{pl}}$: radius of plastic zone: *f*: plastic-zone size factor; *J*: number of dislocation junctions.

	After indent				After retraction			
	$L_{\textrm{disl}}$ (nm)	$R_{\textrm{pl}}$ (nm)	*f*	*J*	$L_{\textrm{disl}}$ (nm)	$R_{\textrm{pl}}$ (nm)	*f*	*J*
Ni	1574	25.6	3.2	308	662	19.9	2.5	178
Fe$_{25}$Ni$_{75}$	1704	33.4	4.2	272	636	22.0	2.8	144
Fe$_{50}$Ni$_{50}$	2030	34.3	4.3	382	764	21.4	2.7	189
Fe$_{75}$Ni$_{25}$	2511	37.1	4.6	489	1508	37.4	4.7	289
Fe	2758	37.5	4.7	479	1810	37.5	4.7	356

Also, the mechanical properties of fcc- Fe$_{50}$Ni$_{50}$ have been measured under uniaxial tension and compared to those of elemental Ni^71^ and it was found that both the yield strength and the ultimate tensile strength of Fe$_{50}$Ni$_{50}$ are superior to that of Ni by roughly a factor of 1.5–2, depending on the measurement temperature. It may be added that the mechanical properties of ternary and quaternary fcc concentrated alloys, such as NiCoCr or FeNiCoCr were found in this study to be superior even to FeNi.

In addition to improved hardness, Fe$_{50}$Ni$_{50}$ also shows improved radiation resistance^72^. A recent experimental study^73^ found similar trends to the ones in our simulations for hardness versus Fe content in Fe$_{x}$Ni$_{1-x}$ alloys, but with a larger increase in hardness compared to Ni, which could be due to the presence of intermetallics in the irradiated samples.

We note that in a recent paper, the simulated compression and tension of Ti$_x$Al$_{1-x}$ and Ti$_x$Ni$_{1-x}$ alloys found maximum strength for equiatomic alloys, $x=0.5$^74^. These alloys are more complex, however, since fcc, bcc and hcp structures compete for varying stoichiometry. On the other hand, the hardness in Ni$_x$Cu$_{1-x}$ increases monotonically with Ni content *x*^75^; in both of these studies^74,75^, no analysis of the USFE was provided. Li *et al.*^76^ use MD simulation to study the change of hardness in Au upon the addition (less than 5%) of dilute alloying elements; they report that hardness scales correlates with the USFE, but even more with the difference of USFE and SFE. We emphasize that in our case of Fe$_{x}$Ni$_{1-x}$ alloys, the correlation of hardness with the difference of USFE and SFE is considerably worse than that with USFE (Fig. 6).

### Dislocation network

**Figure 7:**
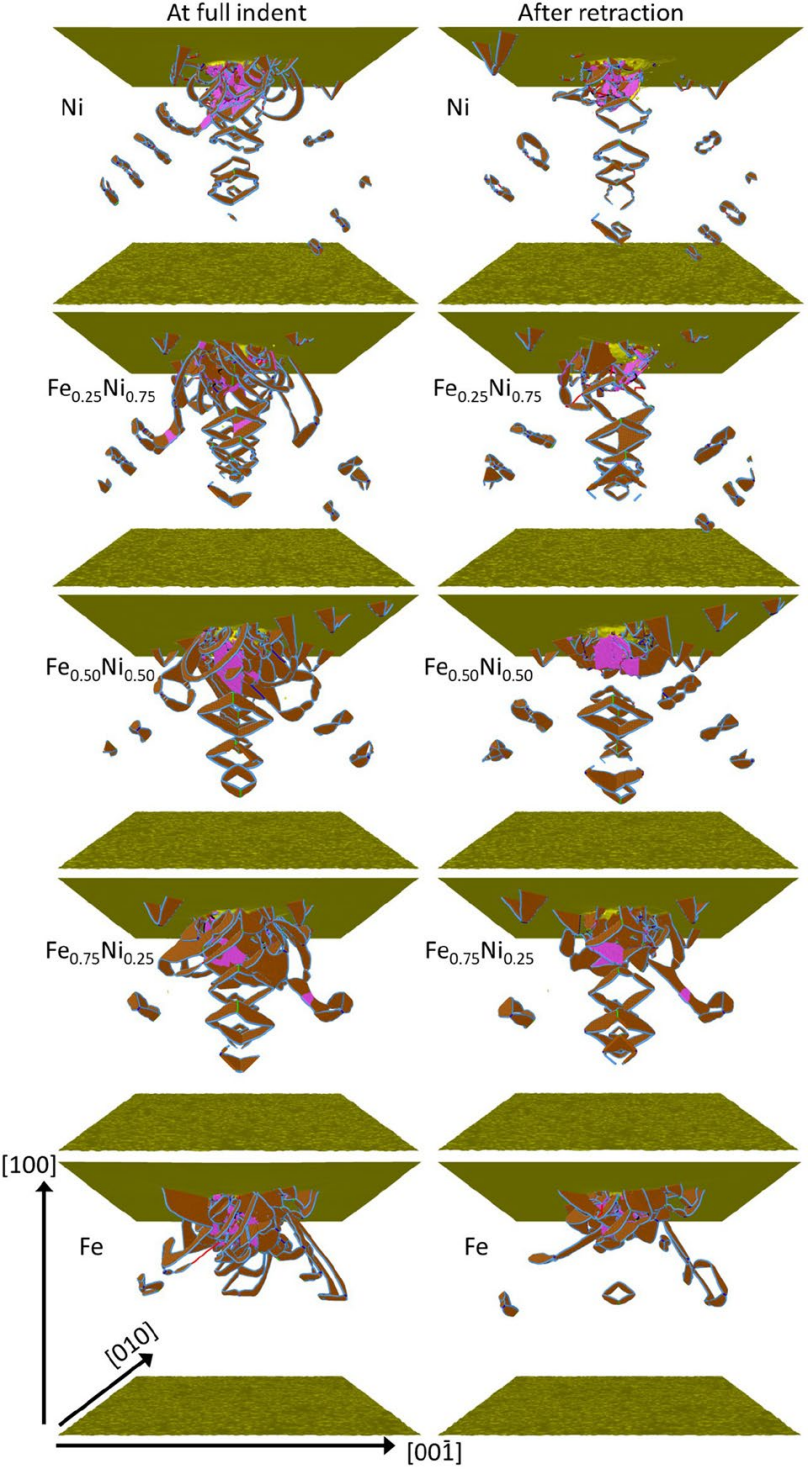
Snapshots showing the stacking-fault planes (brown) and twin boundaries (pink) in elemental Fe and Ni and several random Fe$_{x}$Ni$_{1-x}$ alloys at full indentation (left) and after retraction of the indenter (right). The crystallographic orientations of the sample are indicated in the bottommost panel. Slip occurs on {111} planes in $\langle 110 \rangle$ direction. Twin boundaries are {111} planes and the twin direction is $\langle 112 \rangle$.

During indentation, dislocations are produced in the material. The dislocation network formed during indentation is displayed both immediately at the end of the indentation stage and after retraction of the indenter, see Fig. 7. While elemental Ni features a strong emission of dislocation loops from the plastic zone surrounding the indent pit, addition of Fe suppresses this feature until for pure Fe, only a single loop is found ejected. After retraction of the indenter, the length and number of dislocations evidently shrink. Besides dislocations, also twin boundaries are created by indentation; they are mostly found close to the indent pit in regions of high dislocation density and will be analyzed further in “Twinning”. We note that the loops observed under mechanical deformation are mostly full dislocation loops. This differs from dislocation loops induced by radiation damage, which are mostly bound by partials, with the addition of some stair-rod dislocations bounding stacking fault tetrahedra (SFT) or SFT precursors^21^.

**Figure 8:**
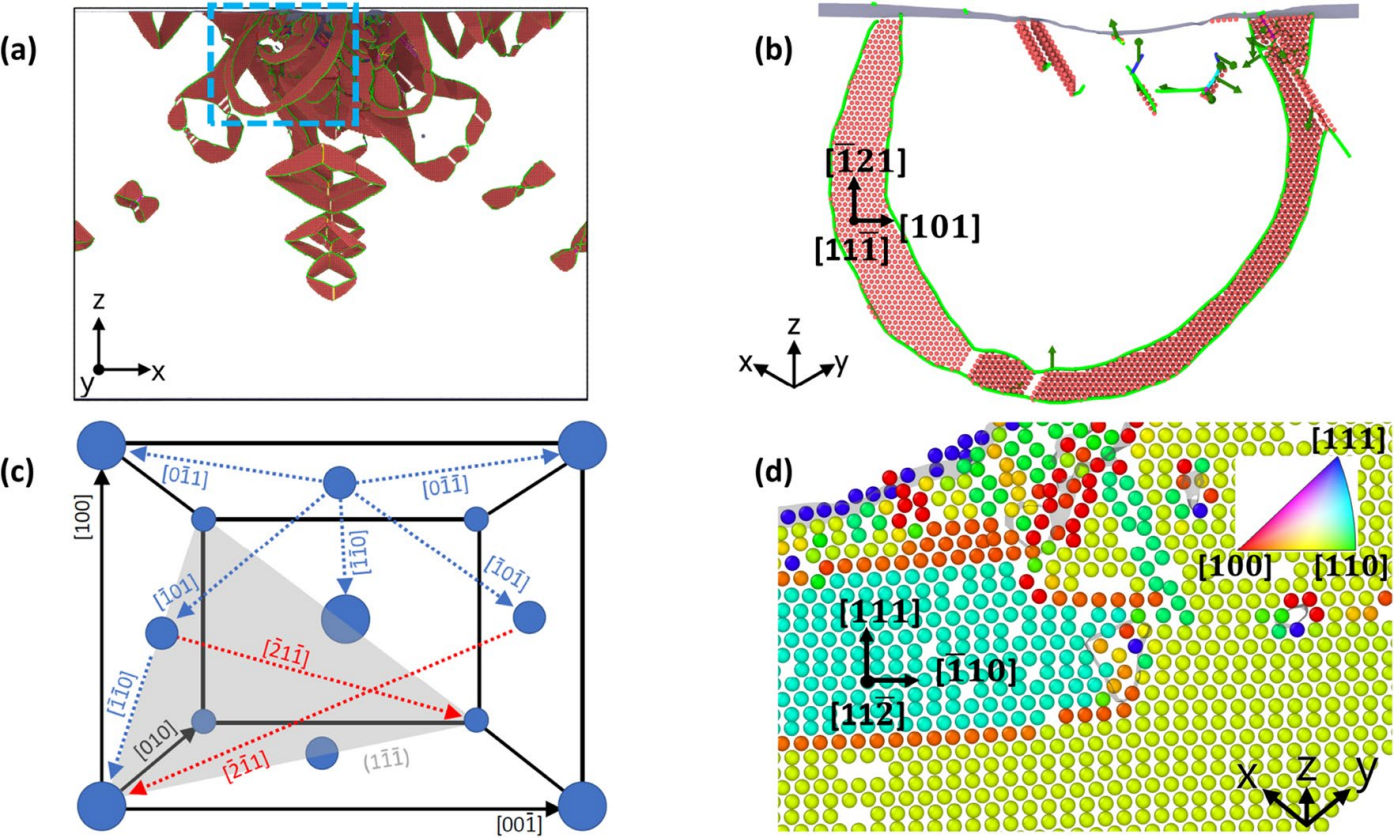
Defect structure in the Fe$_{50}$Ni$_{50}$ crystal after indentation. (**a**) Overview over dislocations; coloring of atoms and dislocation lines as in Fig. 7. (**b**) zooms into the region highlighted by the rectangle in (**a**); note the rotation of the coordinate system. Only a thin slab of thickness 0.2 nm is shown. (**c**) gives a perspective view of an fcc elementary cell with atoms in blue and $\langle 100 \rangle$ directions in black. A (111) plane is highlighted in gray; slip $\langle 110 \rangle$ directions in blue; twin $\langle 112 \rangle$ directions in red. (**d**) shows a twinned region just below the indent pit; atoms are colored according to the local crystal orientation, see insert. The *x*, *y* and *z* directions in all subpanels are aligned with the $\langle 100 \rangle$ directions as in Fig. 7.

Figure 8 exemplifies the character of the dislocations created under indentation for a representative case, the Fe$_{50}$Ni$_{50}$ system immediately after indentation. Figure 8a presents an overview over the defect structure and Fig. 8b zooms into a partial loop that has expanded from the indent pit. As the slab shown is only 0.2 nm thick, only one atomic layer is visible. The dislocation is dissociated into two partials; the stacking fault generated lies in the $(11\bar{1})$ plane. The geometry of the slip systems is shown in Fig. 8c, where besides a (111) plane also several $\langle 110 \rangle$ directions that are relevant in slip are displayed. This figure also shows several twinning $\langle 112 \rangle$ directions. An example of a twinned region is shown in Fig. 8d. The lattice rotation induced by the twinning—around the $[11{\bar{2}}]$ axis perpendicular to the plotted plane—is demonstrated via the coloring of the atoms by the crystal orientation in Fig. 8d. The twin boundary separating the two twin variants appears in orange; it is a {111} plane and the twin direction is $\langle 112 \rangle$. We conclude that plasticity proceeds as expected for fcc materials: Dislocations appear on the {111} planes and glide in $\langle 110 \rangle$ directions^77,78^.

We now discuss quantitatively to what extent the increased alloy hardness is reflected in the properties of the dislocation network. Let us first define the *plastic zone* as the region in which the dislocations generated by the indentation are still connected to the indent pit; this definition deliberatively excludes ejected loops as these are able to move quite freely through the crystal. This definition was made to allow for comparison between simulation results and experimental data^32,79,80^. The plastic zone may be approximated by a hemi-spherical shape, whose radius, $R_{\textrm{pl}}$, is given by the largest distance of a dislocation adjacent to the indent pit to the center of the indentation contact area. The plastic-zone size factor, $f=R_{\textrm{pl}}/a_c$, relates the size of the plastic zone to the contact radius $a_c$. Table 2 quantifies the evolution of these quantities with Fe content *x*. Clearly, the total length of dislocations within the plastic zone continuously increases with Fe content; simultaneously, the size of the zone increases. As Fig. 7 showed, this is caused by the decreased tendency of loop emission for larger Fe content. In fcc-Fe, dislocation cross slip that is necessary to create dislocation loops does not occur leading to the long extended screw segments visible in Fig. 7. Tip retraction causes the plastic zone to recede towards the indent pit for all but the two Fe-richest samples; there the plastic zone remains quite large. The size factors *f* are well in the range of those found for other fcc metals^32,80^, featuring values of $f \lesssim 3$ with the exception of fcc-Fe and Fe$_{75}$Ni$_{25}$, which are exceptionally high due to the lack of dislocation emission.

**Figure 9:**
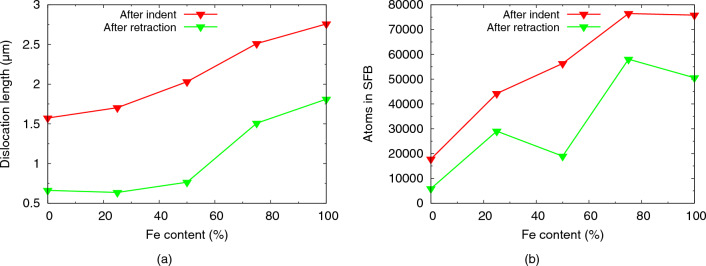
Dependence of the (**a**) dislocation length and (**b**) number of atoms in stacking fault boundaries (SFB) on Fe content in elemental Fe and Ni and in several random Fe$_{x}$Ni$_{1-x}$ alloys.

Figure 9a illustrates the increase of the dislocation length in the plastic zone with Fe content discussed above. The number of atoms contained in stacking faults (Fig. 9b), quantifies the stacking faults created; they follow the same trend. This is plausible, since Shockley partials lead to the formation of stacking faults, cf. the snapshots in Fig. 7, and since Shockley partials form the majority of dislocations, this correlation is valid. The only exception is made by Fe$_{50}$Ni$_{50}$ after tip retraction; here the number of atoms in stacking faults decreases considerably. As Fig. 7 shows, this is caused by a particularly pronounced shrinkage of the plastic zone due to the emission of several loops.

Dislocations in fcc metals are mostly Shockley partials, and our synopsis in Table 3 shows that this is valid also for the series of simulations performed here. During tip retraction, the re-organization of the adjacent network as well as the emission of further loops lets the fraction of Shockley partials shrink. The fraction of sessile dislocations—i.e., stair-rod, Hirth and Frank partials—is thus increased and reaches values > 10% in all cases except pure Fe. For the equiatomic Fe$_{50}$Ni$_{50}$ alloy, the fraction of sessile dislocations is particularly high, reaching almost 20% after tip retraction; this feature agrees well with the high hardness of this alloy.

Dislocation interactions lead to the creation of dislocation junctions within the plastic zone; their numbers are listed in Table 2. A general trend of increasing number of junctions with Fe content can be observed, both before and after tip retraction. This trend is in line with the general increase of dislocation length with Fe content shown in Table 2 and Fig. 9a.

**Figure 10:**
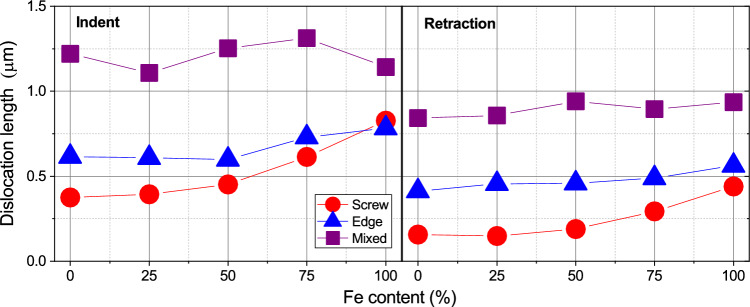
Variation of dislocation type with Fe content.

Finally, dislocation segments can be analyzed according to their screw- or edge-character. To this end, we determine the angle $\theta$ between the Burgers vector and the dislocation line vector of each dislocation segment; we denote a segment as ‘screw’ if the angle is $< 30^\circ$, as ‘edge’ if it is $> 60^\circ$, and as ‘mixed’ otherwise^46^. Figure 10 shows the fraction of screw and edge segments in the entire sample. We observe a general predominance of edge- over screw-type dislocations, with the exception of the pure Fe sample. This corresponds nicely to the large semi-loops shown in Fig. 7 for pure Fe which feature long screw components. For the other samples, the screw components are strongly reduced, since upon pinching off dislocation loops, only the edge component survives. This also explains the strong reduction of the screw component during tip retraction, since the compaction of the dislocation network eradicates screw components. Still, an increase of screw components with Fe content is visible even after tip retraction.

Zhao *et al.*^14^ studied edge dislocation glide in Fe$_{50}$Ni$_{50}$ and compared it to that in pure Ni, using MD simulation based on the Bonny^28^ potential. They find a considerably reduced dislocation glide velocity (at the same shear stress) in Fe$_{50}$Ni$_{50}$ as compared to pure Ni and a strong damping of their motion; the authors argue that this is a characteristic feature of concentrated alloys. Chu *et al.*^81^ showed that screw dislocation glide is less affected. These results do not concern the dislocation semiloops adjacent to the indent pit that form the plastic zone, but rather to the ejected loops that move away from the indentation zone deeper into the crystal.

Hayakawa *et al.*^82^ simulated boxes with a single dislocation dissociated into two partials $\sim$ 7 nm long. They showed that, using the Farkas-Caro potential^83^, FeNi screw dislocations have a smaller core energy than edge dislocations and are hence energetically more stable. They also demonstrated a significantly larger equilibrium separation between edge-type partials for FeNi (3 nm separation) than for Ni (2.2 nm separation). In our simulations, for a complex loading state, we observe more edge than screw dislocations, and many mixed dislocations, as shown in Fig. 10. Qualitatively, partials in Fe display larger separation than partials in Ni, but their separation changes significantly along curved dislocation lines, with values between 1 and 3 nm.

Experimentally, it was found that as-swaged NiFe features an approximately double as high dislocation density than pure Ni^51^; in more complex concentrated alloys, the dislocation density further increases. The increased dislocation density is accompanied by an increase in yield strength.

### Twinning

**Figure 11:**
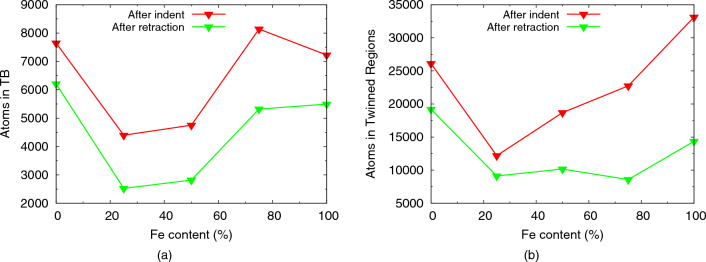
Dependence of the (**a**) number of atoms in twin boundaries (TB), and (**b**) number of atoms in twinned regions on Fe content in elemental Fe and Ni and several random Fe$_{x}$Ni$_{1-x}$ alloys.

The evolution of the atoms in twin boundaries in the plastic zone is displayed in Fig. 11a; in contrast to the dislocation characteristics shown in Fig. 9, twins show a non-monotonic dependence with Fe content. In fact, the number of atoms in twin boundaries strongly decreases from elemental Ni to Fe$_{25}$Ni$_{75}$; this is astonishing since twinning occurs in fcc metals via Shockley partials^84^ and their number is increasing from Ni to Fe$_{25}$Ni$_{75}$, cf. Tables 2 and 3. The high number of atoms in twin boundaries for the Fe-rich species, Fe$_{75}$Ni$_{25}$ and fcc-Fe, is again in line with the general increase of dislocation length for these samples. We emphasize that the mere fact that twinning can be observed in our simulations is non-trivial; simulation studies using smaller samples—such as Ref.^85^ who use only 300,000 atoms—do only observe dislocation formation but no twinning.

In order to obtain a better understanding of the twinning, besides the number of atoms in twin boundaries, we also determined the volume of twinned regions (Fig. 11b), with the method outlined in “Methods”. The figure shows that the twinned volume is maximum for the elemental metals and decreases for alloy samples. Twinning in concentrated alloys requires both large deformation strains and high strain rates^86^; both of these factors are warranted in our nanoindentation simulations. Note that the growth of twinned zones proceeds by the sequential action of Shockley partials moving on adjacent parallel lattice planes in the same direction^84^. Thus twinning requires the easy glide of dislocations in parallel planes; this concerted motion of dislocations is easier in the elemental metals than in the concentrated alloys, where local compositional fluctuations reduce the probability for dislocations to be emitted in two adjacent planes. This feature explains the higher volume of twinned zones in elementary metals as compared to concentrated alloys.

**Figure 12:**
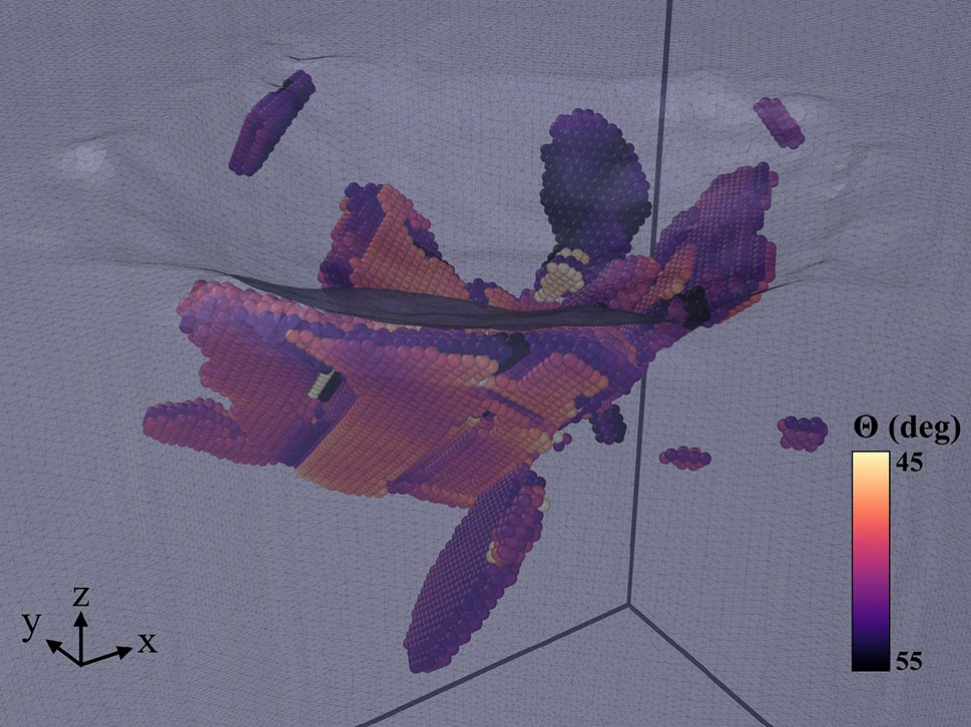
Close-up view of the twinned regions in pure Fe after retraction of the indenter. Atoms are colored by the misorientation, $\theta$, towards the original lattice. A surface mesh allows to identify the indent pit.

A snapshot featuring the volumetric aspect of twinning in our simulations is given in Fig. 12 for the case of pure Fe. Around the indent pit, several regions are visible where the width of the twinned regions has reached up to 10 layers.

By comparing the data for twin boundaries and twin volumes in Fig. 11a,b, the ratio of atoms in the twinned regions surpasses that of the atoms in twin boundaries by a factor of 10 or more for pure iron, but decreases towards the Ni-rich alloys and assumes values of only 1–2 for pure Ni. This corresponds to the high thickness of the twinned regions in pure Fe of up to 10 monolayers.

### Surface imprints

**Figure 13:**
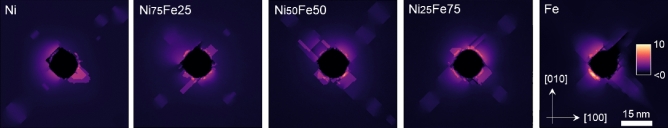
Top view of the residual surface imprints for the samples investigated. Color scale indicates height above the original surface, in Å units.

A standard practice to analyze nanoindentation experiments is the exploration of the indented surface by means of atomic force microscopy (AFM). Figure 13 shows the structure of the residual imprints produced on the surface of each sample. While the expected four-fold symmetry for (100)-oriented fcc metals is in general visible, notable features can be highlighted. While a circular imprint introduced by the spherical indenter is expected, our results appear to have a square form. This effect has been reported before^87^ and was attributed to hardening effects.

Pure Fe exhibits a pile-up which shows the highest hillocks; the average pile-up height decreases with the decreasing Fe content, with Ni having the lowest pile-up height. Fe displays a notable sink-in on the top-right quadrant. Experiments and crystal plasticity studies demonstrated that a high strain-hardening potential is the cause for a tendency to sink-in^88^. Indeed, the Fe sample displays both the largest dislocation length and the largest amount of dislocation junctions, cf. Table 2, and can therefore be assumed to have the highest potential for strain-hardening. We also note that the pressure drop during the hold phase (Fig. 4), is smallest for pure Fe, suggesting less plastic relaxation and more strain-hardening for Fe than in the other materials. For the Fe$_{x}$Ni$_{1-x}$ binary alloys, the pile-ups do show some changes from one composition to the other and the four-fold symmetry is indeed less clear. This correlates with the fact that the binary nature of the alloys is expected to introduce local compositional fluctuations, influencing slip-system activation, dislocation evolution and also twinning^89^, as described in the previous sections. We do not observe significant differences in the imprint profiles, as chemical disorder is maximized for the Fe$_{x}$Ni$_{1-x}$ samples with respect to the pure Fe or Ni samples. This is different from the role of chemical disorder in high-entropy alloys, which contributes to higher imprint isotropy^90,91^.

## Summary

Using molecular dynamics simulation, we studied the indentation of single-phase fcc Fe$_{x}$Ni$_{1-x}$ alloys ($x=0.25$, 0.5 and 0.75) and compared it to the behavior of the pure elements, Ni and fcc-Fe. By using sufficiently large simulation samples, including around 20 million atoms, the plastic processes occurring under the indenter—dislocation loop emission as well as build-up and growth of twinned zones—could be described in detail. We obtained the following main findings.

1. The indentation hardness is highest for the equiatomic alloy, $x=0.5$. This finding is in agreement with experimental results on the strength of these alloys under uniaxial tensile strain^71^.
2. The high strength of Fe$_{50}$Ni$_{50}$ can be rationalized by the unstable stacking fault energy of this alloy, which maximizes at this stoichiometry. Since the USFE quantifies the barrier to dislocation glide, the USFE correlates with hardness^60–62^.
3. While pure Ni features strong emission of dislocation loops from the plastic zone under the indenter—and a concomitantly small plastic zone adjacent to the indent pit—loop emission recedes and the plastic zone increases with Fe content. Simultaneously, the length of the dislocation network and the number of atoms in stacking faults generated in the plastic zone increase.
4. With increasing Fe content, also the fraction of screw segments in the plastic zone increases. The smaller mobility of screw dislocations explains qualitatively the larger plastic zone for Fe-rich samples.
5. We find a considerable contribution of twinning in the plastic zone. The volume of twinned regions in the plastic zone is highest for the elemental solids but decreases for the alloys. This feature is explained by the fact that twinning proceeds by the glide of dislocations on adjacent parallel lattice planes; this concerted motion is less efficient in the alloys.
6. Surface imprints show increasing pile-up heights with increasing Fe content.

Our finding that the hardness of single-phase alloys follows the USFE might be useful in designing new materials with particular emphasis on their hardness properties. Techniques such as ion implantation or plasma deposition allow to create gradient profiles in stoichiometry which then translate into gradient hardness profiles. Other applications of our findings may use the embedding of precipitates of prescribed hardness into a matrix material; for Fe$_{x}$Ni$_{1-x}$ alloys, the preparation of such materials is eased by the fact that the lattice constants do not vary much with stoichiometry *x*.

**Table 3:** Length of dislocations in the plastic zone as relative values after indent and after retraction. Data are given as fractions (in percent) of the total dislocation length (Table 2). Mobile dislocations: Shockley partials with Burgers vector $1/6\langle 112 \rangle$ and perfect $1/2\langle 110 \rangle$ dislocations. Sessile dislocations: stair-rod partials $1/6\langle 110 \rangle$, Hirth partials $1/3\langle 001 \rangle$, and Frank partials $1/3\langle 111 \rangle$.

		Shockley	Perfect	Stair-rod	Hirth	Frank	Unidentified
Ni	Indent	85	3	4	1	1	6
	Retract	70	5	7	1	4	13
Fe$_{25}$Ni$_{75}$	Indent	85	1	4	2	1	7
	Retract	70	7	10	1	2	10
Fe$_{50}$Ni$_{50}$	Indent	80	1	8	3	1	7
	Retract	68	3	13	4	2	10
Fe$_{75}$Ni$_{25}$	Indent	79	1	6	4	< 1	9
	Retract	79	1	9	5	< 1	6
Fe	Indent	81	3	3	4	1	10
	Retract	81	1	4	4	1	9

An experimental verification of our prediction that dislocation semiloops become more extended and the plastic zone grows with increasing Fe content might be particularly straightforward in nano-pillars cut out from single-phase Fe$_{x}$Ni$_{1-x}$ alloys, but should also be possible in large single-crystalline grains as it was recently done in austenitic steel^92^. Since the size of the plastic zone scales with the radius of the contact zone and thus with the indenter size, using a larger indenter will emphasize the size differences in the plastic zones for varying stoichiometry *x*.

## Data Availability

All data used for this study are contained in this article.
